# Uncoupling protein 4 (*UCP4*) gene variability in neurodegenerative disorders: further evidence of association in Frontotemporal dementia

**DOI:** 10.18632/aging.101632

**Published:** 2018-11-13

**Authors:** Alberto Montesanto, Paolina Crocco, Serena Dato, Silvana Geracitano, Francesca Frangipane, Rosanna Colao, Raffaele Maletta, Giuseppe Passarino, Amalia C. Bruni, Giuseppina Rose

**Affiliations:** 1 Department of Biology, Ecology and Earth Sciences, University of Calabria, Rende, Italy; 2 Regional Neurogenetic Centre, ASP CZ, Lamezia Terme, Italy; * Equal contribution

**Keywords:** Frontotemporal dementia, Parkinson disease, APOE, UCP4, uncoupling proteins, mitochondria, neurodegeneration

## Abstract

Ongoing research suggests that mitochondrial dysfunction is a common hallmark in neurodegenerative diseases, pointing to mitochondrial uncoupling process as a critical player. We recently reported that rs9472817-C/G, an intronic variant of neuronal mitochondrial uncoupling protein-4 (*UCP4/SLC25A27*) gene affects the risk of late onset Alzheimer's disease (LOAD), and that the variant's effect is strongly dependent on *APOE*-ε4 status. Here, we extended our analysis to a cohort of 751 subjects including late-onset familial and sporadic cases of frontotemporal dementia (FTD; 213), Parkinson disease (PD;96), and 442 healthy controls. In all subgroups, carriers of *APOE*-ε4 allele were at higher risk of disease. Regarding the rs9472817, no association was detected in familial FTD and both subgroups of PD patients. In sporadic FTD, as in LOAD, we found that the C allele increased the risk of disease of about 1.51-fold in a dose-dependent manner (p=0.013) independently from that conferred by *APOE*-ε4. Expression quantitative trait loci (eQTL) data of different brain regions suggest that rs9472817 likely exerts its effect by a cis-regulatory mechanism involving modulation of *UCP4*. If validated, the involvement of UCP4 in both FTD and LOAD might indicate interesting shared etiological factors which might give future therapeutic clues.

## Introduction

Neurodegenerative diseases comprise one of the major public health concerns worldwide, with Alzheimer’s disease (AD), Frontotemporal disease (FTD), and Parkinson’s disease (PD) being the most common types. Although these diseases are considered distinct entities, each with its distinct etiological mechanisms, affected brain regions and clinical characteristics, they share some common features. For instance, some behavioral disturbances can characterize the initial phases of AD patients, while some FTD patients can manifest deficit in the episodic memory domain [1]. From a neuropathological point of view, accumulation of intracellular tau protein is seen in both AD and FTD [2]; moreover, TAR-DNA binding protein (TDP)-43 deposition has been reported in AD, in a subtype of FTD and, in some rare PD cases, associated with a leucine-rich repeat kinase 2 (*LRRK2*) mutation [3,4]. Evidence also point to potential genetic overlap among these disorders [5]. The *MAPT* gene, extensively investigated in FTD [2], has been also implicated in AD and PD [6,7]. In addition, evidences suggest that genetic variants within the HLA locus contribute to the development of these disorders [8–10]. Taken together, such overlapping features may reflect some common underlying etiological factors. 

The impairment of mitochondrial functioning is now emerging as an upstream event in the chain of pathological events leading to neuronal degeneration and a shared feature of these neurodegenerative diseases [11–15]. What is more, mitochondrial dysfunction, oxidative stress and abnormal accumulation of misfolded and aggregated proteins seem to be interdependent phenomena that work in concert, reinforcing each other to drive these pathologies, as summarized in two recent reviews [16,17]. An inherent part of mitochondrial physiology which could impact on neuronal functioning is the uncoupling of respiration from oxidative phosphorylation, a process mediated by three out of five mitochondrial uncoupling proteins (UCP2,4,5) which affects energy production, oxidative stress and intracellular calcium homeostasis [18,19].

We recently found that a non-coding variant (rs9472817) in the last intron of *UCP4/SLC25A27* gene affects the risk of developing sporadic and familial late onset Alzheimer's disease (LOAD) and strongly modulates the effect of *APOE*-ε4 on the disease risk [20]. *UCP4*, which is prominently expressed in neurons from hippocampus, cortex, Substantia nigra pars compacta, striatum and cerebellum [21,22] and, at lower levels, in astrocytes [23], appears to have a critical role in helping neurons to cope with conditions of metabolic and oxidative stress [22]. Thus, it is reasonable to hypothesize that UCP4 genotypes may foster the development of multiple neurodegenerative conditions.

In order to test this hypothesis, we investigated whether the rs9472817 variation of *UCP4* gene also influences the risk of developing FTD and PD and whether this eventual risk is modulated by the *APOE*-ε4 genotypic variability.

## RESULTS

**Figure 1:**
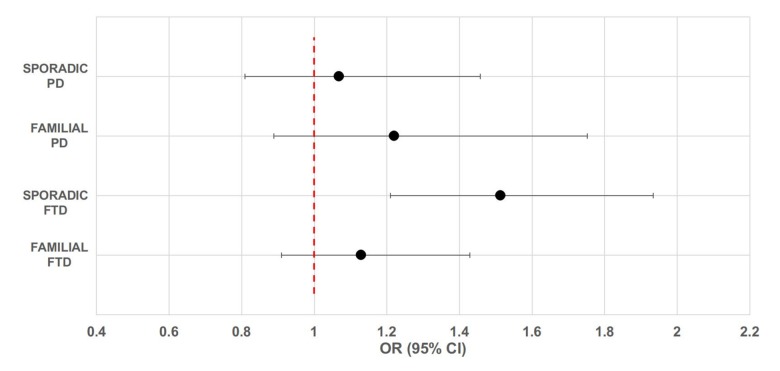
**Forest plot of overall analysis for the association between rs9472817 in UCP4 and risk of familial and sporadic FTD and PD.** The circle and horizontal lines represent odds ratio (OR) and 95% confidence interval (CI).

Descriptive information about participants is presented in Table 1. It is of note that there was no difference in age and gender between patients and healthy controls. Table S1 reports the allele and genotype frequencies for the *UCP4*-rs9472817 in patients and controls. Genotype distributions were in agreement with the HW equilibrium (P-value > 0.05). We did not detect any significant effect of the rs9472817 polymorphism on the risk of sporadic and familial PD and familial FTD (Figure 1). On the contrary, our results suggest that *UCP4* may be a susceptibility gene for sporadic FTD. The data from this subgroup of patients showed, as previously reported for LOAD [20], that individuals with one or more copies of the C allele for rs9472817 are at increased risk of disease compared to those with the GG genotype, with a per-allele increased risk of 1.513 (95% CI 1.091-2.098, P-value= 0.013). After adjusting for possible confounders, this risk resulted almost unchanged (OR=1.506, 95% CI 1.085-2.090, P-value=0.014). We also evaluated the potential association of the UCP4 rs9472817-C allele with the disease progression and severity, as measured by age of disease’s onset and MMSE values We did not find statistically significant difference between carriers and non-carriers of the C allele according to age of disease’s onset and MMSE scores. We found that the presence of the *APOE*-ε4 allele was associated with an increased risk of FTD, both in familial (OR= 6.341, 95%CI: 3.747-10.731, P-value=5.97*10^-12^) and sporadic cases (OR= 3.621, 95%CI: 2.027-6.470, P-value=1.4*10-5), as well as in the familial (OR=4.764, 95%CI: 2.205-10.294, P-value=7.2*10^-5^) and in the sporadic form of PD (OR= 2.552, 95%CI: 1.171-5.562, P-value=0.018) (Table 2, model 1). However, the reported effect of *UCP4*-rs9472817 variation on sporadic FTD remained statistically significant also after adjustment for *APOE*-ε4 status (OR=1.599, 95%CI: 1.135-2.252, P-value=0.007, model 2). In other words, in sporadic FTD, the *UCP4*-rs9472817 variation affects the risk of dementia independently of the presence of *APOE*-ε4 allele. Furthermore, interaction analysis enabled us to verify that in these models, the interaction terms were not significantly different from 0 (model 3).

**Table 1:** Characteristics of the analyzed sample in cases and controls.

	**FTD**		**Parkinson**		**Controls** **(N=442)**
	**Familial** **(N=113)**	**Sporadic** **(N=100)**	**Familial** **(N=41)**	**Sporadic** **(N=55)**	
Age (mean ± SD)	73.9 ± 6.8	75.2 ± 6.9	74.9 ± 6.8	74.8 ± 6.9	73.7 ± 8.8
Males [n (%)]	49 (43.4)	43 (43.0)	23 (56.1)	29 (52.7)	225 (50.9)
Age onset (mean ± SD)	72.9 ± 5.3	72.5 ± 5.7	66.7 ± 6.4	67.0 ± 8.2	-
MMSE^1^ (mean ± SD)	11.2 ± 8.1	13.1 ± 7.5	24.1 ± 5.2	23.8 ± 5.4	23.5 ± 4.2
*APOE*-ε4 carriers [n (%)]	42 (37.2)	25 (25.3)	12 (30.8)	10 (19.2)	32 (8.5)

^1^MMSE scores were adjusted for educational level and age at inclusion

**Table 2:** Results of the logistic regression models for FTD and PD genetic risk.

			**Familial**			**Sporadic**		
**Group**	**Model**		**OR**	**95% CI**	**P-value**	**OR**	**95% CI**	**P-value**
**FTD**	Model 1	APOE-ε4	6.341	3.747-10.731	5.97*10^-12^	3.621	2.027-6.470	1.4*10^-5^
							
	Model 2	APOE-ε4	6.598	3.776-11.530	3.45*10^-11^	3.557	1.917-6.559	5.7*10^-5^
		UCP4-rs9472817	1.293	0.916-1.825	0.144	1.599	1.135-2.252	0.007
							
	Model 3	APOE-ε4	4.888	1.827-13.078	0.002	3.189	1.067-9.535	0.038
		UCP4-rs9472817	1.207	0.817-1.783	0.346	1.567	1.071-2.292	0.021
		APOE-ε4*UCP4-rs9472817	1.357	0.592-3.112	0.471	1.111	0.465-2.657	0.813
**PD**			**Familial**			**Sporadic**		
	**Model**		**OR**	**95% CI**	**P-value**	**OR**	**95% CI**	**P-value**
	Model 1	APOE-ε4	4.764	2.205-10.294	7.2*10^-5^	2.552	1.171-5.562	0.018
							
	Model 2	APOE-ε4	5.255	2.376-11.624	4.2*10^-5^	2.082	0.889-4.880	0.091
		UCP4-rs9472817	1.314	0.795-2.172	0.288	1.162	0.756-1.786	0.494
							
	Model 3	APOE-ε4	3.572	0.859-14.848	0.080	2.138	0.537-8.511	0.281
		UCP4-rs9472817	1.182	0.655-2.134	0.579	1.167	0.731-1.863	0.517
		APOE ε4*UCP4-rs9472817	1.447	0.473-4.427	0.518	0.972	0.295-3.200	0.962

## DISCUSSION

FTD, PD and LOAD are clinically distinct conditions although they share similar dysfunctional phenotypes, such as protein aggregation, neuronal cell death, and cognitive decline. Yet, the extent of the genetic overlap between these disorders is still not fully known, despite considerable research to date. Assessing genetic overlap between complex traits is based on the notion that genes or genetic variants may play a role in related phenotypes in the context of different genetic backgrounds and under different environmental conditions because of their pleiotropic functions [24].

Building on our prior work implicating the involvement of the rs9472817 in *UCP4* gene in LOAD [20], in the current study we assessed the potential pleiotropic effect of this variant on late-onset familial and sporadic FTD and PD. Our data suggest that this SNP is a marker also for sporadic FTD. The lack of association with familial FTD suggests that this variant probably confers a low risk of disease, which could be covered by major risk factors involved in the familial form of the disease. On the other hand, association studies indicate that sporadic FTD is a polygenic trait, arising from the influences of multiple pleiotropic loci with small individual effects [5], likely including *UCP4* gene.

As we stated previously, FTD and LOAD are characterized by impairment in different cognitive domains. However, there are levels of overlap between these disorders, i.e. some behavioral disturbances can characterize the initial phases of AD patients, while some FTD patients can manifest deficit in the episodic memory domain, that likely reflect the fact that FTD and AD are associated with progressive impairment of similar brain circuits [1]. This is suggestive of a potential pathological overlap and of the existence of common genetic mechanisms that drive the pathological events leading to their onset. Thus, our data may provide new insights into the underlying shared pathogenic mechanisms between FTD and LOAD.

No association was detected between rs9472817 and PD, indicating that this genetic factor is likely not implicated in the development of this type of dementia. However, given the reduced number of PD patients evaluated in the present study, this could be also due to a type II error and thus, a role of rs9472817 on disease risk, even if small, cannot be rule out.

We assessed the functional consequences of the rs9472817 on gene expression by querying three different brain-specific expression quantitative trait loci (eQTLs) datasets (see Supplementary Table S2 and Figure S1). Data are consistent regarding an increased expression of *UCP4* in different brain regions associated to the presence of the risk C allele of rs9472817, possibly indicating that higher levels of UCP4 might be the mechanism by which the association at this locus is mediated. In terms of biological plausibility this would seem to be not in accordance with the effect we found on the risk of FTD, since experimental evidences document that increased levels of UCP4 allow to preserve the physiological functions of neurons by maintaining energy and redox balance and by decreasing mitochondrial calcium accumulation [21,25,26]. On the other hand, the increased expression of *UCP4* associated to the C allele is in accordance with our previous study showing that this is a longevity allele [27]. The reasons for these contrasting results are not clear, however it can be hypothesized that a phenomenon of genetic epistasis (the phenotypic effect of the allele depends on the specific alleles at another locus) is present or that the phenotypic effect of the allele vary depending on cell’s biological context. As for the latter case, for example, evidence indicate that UCP4 acts as a buffering mechanism that finely tunes the entry of cytosolic Ca2+ into the mitochondria, avoiding the calcium overload in the organelle. In other conditions, such as in neurodegenerative diseases where increased levels of cytosolic Ca2+ have been documented [28–30], an increased expression of *UCP4*, reducing the uptake of Ca2+ in mitochondria might worsen the cytosolic overload, further accruing the pathological accumulation of this element.

It must however be pointed out that, according to eQTL data, the risk C allele is also significantly associated with decreased *TDRD6* expression in brain tissues. This gene encodes a tudor domain-containing protein, a male germ line-specific protein involved in spermiogenesis, chromatoid body formation, regulation of miRNA expression and the nonsense mediated decay pathway [31,32]. To our knowledge, there is no evidence to support a link between TDRD6 and FTD or other neurodegenerative diseases. Therefore, although a role cannot be ruled out, it seems unlikely that alterations in the levels of this gene might be the molecular basis of the observed associations, and this deserves further attention.

With respect to the involvement of APOE in FTD and PD, results in literature are inconsistent regarding the effect of the ε4 allele: some studies report an increased risk associated to this allele whereas others report no association [33–36]. In particular, studies focusing on the role of APOE in PD remain largely inconclusive, with some studies reporting *APOE*-ε2 allele associated with higher prevalence of sporadic PD [37]. Here we found that in all subgroups analyzed, carriers of *APOE*-ε4 allele are at higher risk of disease. In our previous study on LOAD, we observed a strong interaction between rs9472817 genotype and *APOE*-ε4 in determining the disease risk [20]. Here, we did not observe any significant interactions. However, subjects carrying at least one APOE-ε4 allele combined with the rs9472817-CC genotype had a significantly higher risk of sporadic FTD. The lack of interaction may be a consequence of an insufficient sample size, and hence the power to unveil a small interaction effect is limited, or it may be attributed to the lower risk conferred by the *APOE*-ε4 to FTD [38]. Alternatively, and more simply, it may be due to the fact that in FTD there is no interaction between these variants.

There are some limitations to our study. First, the relatively small size of our sample makes it necessary to replicate the current result with a larger sample and, possibly, in other countries. It should be noted however, that this study was designed to replicate our previous data implicating the involvement of *UCP4*-rs9472817 with LOAD. Furthermore, we did not provide experimental evidence in support of the hypothesis that the detrimental effect of the C allele may depend on the increased expression of *UCP4*, so functional studies in this sense should be carried out. Although our study does not allow definitive conclusions, data here presented can be a source of inspiration for future studies, to better clarify the role of mitochondrial uncoupling in the pathogenesis of neurodegenerative diseases and the possible causative mechanisms at the origin of these disorders.

## METHODS

### Subject selection

Unrelated patients with late-onset FTD (N=213) and PD (N=96) were recruited at the Regional Neurogenetic Centre (Calabria, southern Italy). Patients without other affected members in the family were classified as sporadic case (100 subjects with FTD and 55 subjects with PD), while patients with a positive family history of disease were classified as familial case (113 subjects with FTD and 41 subjects with PD) (Table 1).

A control group of 442 unrelated healthy subjects matched for age, sex and ethnicity was recruited in the same population.

The study was approved by the local ethics committee and conducted in accordance with the provisions of the Helsinki Declaration and a written informed consent was obtained from all individuals involved in the study; for disabled patients consent was given by their legal tutors.

### Clinical assessment

Diagnosis of FTD was assessed by using multiple operational criteria and was based on specific clinical neuropsychological features and neuro-radiological profiles according to the Neary criteria [39] or the revised criteria for behavioural FTD [40]. No known pathological mutations of *MAPT* (microtubuleassociated protein tau gene), *GRN* (progranulin) and *C9orf72* (chromosome 9 open reading frame 72) were detected in FTD patients. Diagnosis of Parkinson's disease was ascertained according to Gelb’s diagnostic criteria for PD [41].

The same complete set of clinical-laboratory procedures and neurological assessment of cognitive status used for patients were also performed in the control group.

### Genotyping and statistical analysis

Genotyping and statistical analysis of the data were performed as previously described [20]. Briefly, the genotype of the *UCP4*-rs9472817 SNP was determined by Sequenom iPLEX Gold platform. Genotyping of the two SNPs, rs439358 and rs7412, used to determine *APOE* genotype was conducted according to the protocol described in [42]. Allele and genotype frequencies were estimated by gene counting from the observed genotypes. Hardy–Weinberg (HW) equilibrium was tested by Fisher’s exact test. The association between the analyzed genetic variants and the disease phenotype was assessed by fitting logistic regression models. In these models genotypes at the *UCP4*-rs9472817 polymorphism were coded in an additive fashion (number of copies of the minor allele), while those at *APOE* locus were coded in a dominant fashion (carrier/non carrier of ε4 allele). The same model was also used for evaluating possible gene-gene interactions on the disease susceptibility. In such models, age, gender, MMSE score and age of disease’s onset were also used as confounder factors.

## Supplementary Material

Supplementary Material
