# Supplementary material for: Uncoupling protein 4 (UCP4) gene variability in neurodegenerative disorders: further evidence of association in Frontotemporal dementia
Source: Aging (Albany NY). 2018 Nov 13;10(11):3283–93. doi: 10.18632/aging.101632 (PMC6286830; doi:10.18632/aging.101632)
Supplement: Supplementary Material [file aging-10-101632-s001.pdf]

SUPPLEMENTARY MATERIAL

SUPPLEMENTARY FIGURE

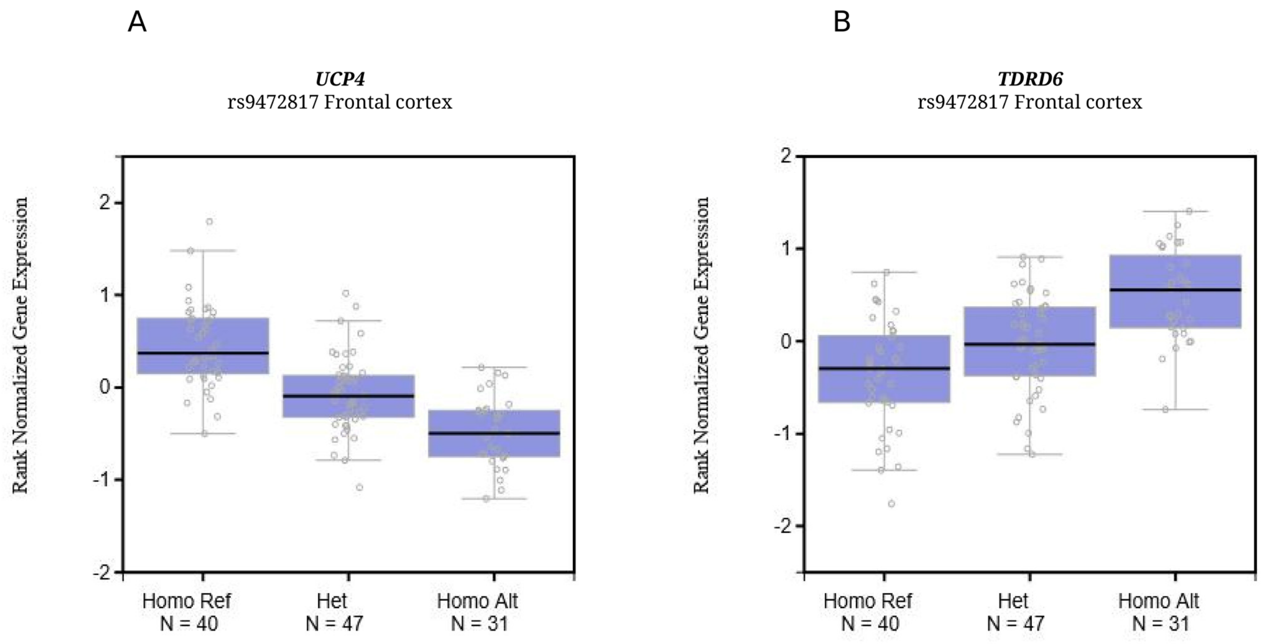

Figure S1. Expression quantitative trait loci (eQTL) box plots of associations between genotypes of rs9472817 with UCP4 (**A**) and TDRD6 (**B**) expression in frontal cortex from the Genotype-Tissue Expression (GTEx) database. X-axes represent the genotypes of the SNP, with the sample size (N) in each group indicated below. Ref stands for reference allele (C), and alt stands for the alternate allele (G). Y-axes represent gene expression levels obtained from RNA-seq. Error bars indicate the standard error of the mean.

SUPPLEMENTARY TABLES

Table S1. Distribution of allelic and genotypic frequencies of *UCP4*-rs9472817 in patients and controls.

**Allele**	**Familial FTD**	**Sporadic FTD**	**Familial PD**	**Sporadic FTD**	**Controls**
C	112 (57.1)	122 (64.2%)	46 (59.0%)	58 (55.8%)	445 (54.1%)
G	84 (42.9%)	68 (35.8%)	32 (41.0%)	46 (44.2%)	377 (45.9%)
**Genotypes**					
C/C	34 (34.7%)	42 (44.2%)	14 (35.9%)	18 (34.6%)	119 (29.0%)
C/G	44 (44.9%)	38 (40.0%)	18 (46.2%)	22 (42.3%)	207 (50.4%)
G/G	20 (20.4%)	15 (15.8%)	7 (17.9%)	12 (23.1%)	85 (20.7%)
**HWE (P-value)**	0.414	0.262	0.749	0.397	0.842

Table S2. eQTL analysis of the rs9472817-C/G in different brain regions, as retrieved by three eQTL databases (GTEx, LIBD Brainseq and Braineac).

eQTL Database	Tissue	Gene symbol	Effect size	p-value
GTEx				
	Amygdala	SLC25A27	-0.56	$7.0e^{-9}$
	Anterior cingulate cortex (BA24)	SLC25A27	-0.47	$4.8e^{-10}$
	Caudate (basal ganglia)	TDRD6	0.69	$4.0e^{-17}$
		SLC25A27	-0.43	$5.8e^{-14}$
	Cerebellar Hemisphere	SLC25A27	-0.71	$5.6e^{-16}$
		TDRD6	0.51	$2.0e^{-8}$
	Cerebellum	TDRD6	0.64	$8.3e^{-18}$
		SLC25A27	-0.62	$9.5e^{-16}$
	Cortex	SLC25A27	-0.63	$1.9e^{-15}$
		TDRD6	0.53	$5.3e^{-11}$
	Frontal Cortex (BA9)	SLC25A27	-0.62	$6.4e^{-19}$
		TDRD6	0.56	$7.8e^{-11}$
	Hippocampus	SLC25A27	-0.62	$1.5e^{-11}$
		TDRD6	0.47	$1.9e^{-8}$
	Hypothalamus	SLC25A27	-0.53	$8.7e^{-9}$
		TDRD6	0.53	$1.1e^{-6}$
	Nucleus accumbens (basal ganglia)	SLC25A27	-0.63	$4.1e^{-16}$
		TDRD6	0.48	$8.9e^{-8}$
	Putamen (basal ganglia)	SLC25A27	-0.50	$1.3e^{-10}$
		TDRD6	0.64	$6.9e^{-10}$
	Spinal cord (cervical c-1)	SLC25A27	-0.44	$1.1e^{-6}$
	Substantia nigra	SLC25A27	-0.52	$4.1e^{-9}$
LIBD Brainseq	Dorsolateral prefrontal cortex	SLC25A27	-0.10, -0.16	$< 8.6e^{-7}$
		TDRD6	0.10, 0.29	$< 4.1e^{-5}$
Braineac	Cerebellum	SLC25A27, TDRD6	-	$< 1.1e\text{-}^{1}$
	Frontal Cortex	SLC25A27, TDRD6	-	$< 1.7e^{-1}$
	Hippocampus	SLC25A27, TDRD6	-	$< 1.5e^{-1}$
	Intralobular white matter	SLC25A27, TDRD6	-	$< 1.6e\text{-}^{1}$
	Medulla oblongata	SLC25A27, TDRD6	-	$< 1.3e^{-1}$
	Occipital Cortex	SLC25A27, TDRD6	-	$< 3.5e^{-1}$
	Putamen	SLC25A27, TDRD6	-	$< 1.1e^{-1}$
	Substantia Nigra	SLC25A27, TDRD6	-	$< 1.8e^{-1}$
	Temporal Cortex	SLC25A27, TDRD6	-	$< 1.2e^{-1}$
	Thalamus	SLC25A27, TDRD6	-	$< 1.9e\text{-}^{1}$

The effect size refers to the minor allele (G) and provides the variation in the strength of expression. Positive numbers indicate higher mRNA levels in samples carrying the minor allele compared to those with the major allele, while negative numbers indicate lower mRNA levels in samples with the minor allele. In LIBD, a range is available for each gene. For each association found, the p-value is reported, as unique value or as minimum of several observations, when different transcripts of the gene where tested.

SUPPLEMENTARY METHODS

Methodology

The possible functional effect of rs9472817 was assessed by using data from RNA-Seq where genotypes and expression levels are assayed for a large number of individuals allowing to identify expression quantitative trait loci (eQTLs) in non-coding regions. Firstly, we referred to data from GTEx (Genotype–Tissue Expression) dataset (https://gtexportal.org/), a comprehensive survey of the functional consequences of genetic variation at the transcript level from various human tissues samples [1]. Then, we also used two other relevant brain-specific eQTL datasets: the Lieber Institute for Brain Development (LIBD) RNA-Seq data, accessed via the LIBD eQTL browser at http://eqtl.brainseq.org, which includes data from the dorsolateral prefrontal cortex, DLPFC) of schizophrenia patients and controls [2], and the Braineac (http://www.braineac.org) dataset from the UK Brain Expression Consortium (UKBEC) which includes brain regions from individuals free of neurodegenerative disorders analysed using the Affymetrix Exon 1.0 ST Array [3].

SUPPLEMENTARY REFERENCES

1. The Genotype-Tissue Expression (GTEx) project. Nat Genet. 2013; 45: 580-5. https://doi.org/10.1038/ng.2653

2. BrainSeq: A Human Brain Genomics Consortium. BrainSeq: Neurogenomics to Drive Novel Target Discovery for Neuropsychiatric Disorders. Neuron. 2015; 88:1078-83. https://doi.org/10.1016/j.neuron.2015.10.047

3. Ramasamy, A. et al. Genetic variability in the regulation of gene expression in ten regions of the human brain. Nat Neurosci. 2014; 17: 1418–28. https://doi.org/10.1038/nn.3801
